# Training Load and Fatigue Marker Associations with Injury and Illness: A Systematic Review of Longitudinal Studies

**DOI:** 10.1007/s40279-016-0619-5

**Published:** 2016-09-28

**Authors:** Christopher M. Jones, Peter C. Griffiths, Stephen D. Mellalieu

**Affiliations:** 1 Research Centre in Applied Sports, Technology, Exercise and Medicine, College of Engineering, Swansea University, Fabian Way, Swansea, SA1 8QQ Wales, UK; 2 Cardiff School of Sport, Cardiff Metropolitan University, Cardiff, Wales, UK

## Abstract

**Background:**

Coaches, sport scientists, clinicians and medical personnel face a constant challenge to prescribe sufficient training load to produce training adaption while minimising fatigue, performance inhibition and risk of injury/illness.

**Objective:**

The aim of this review was to investigate the relationship between injury and illness and longitudinal training load and fatigue markers in sporting populations.

**Methods:**

Systematic searches of the Web of Science and PubMed online databases to August 2015 were conducted for articles reporting relationships between training load/fatigue measures and injury/illness in athlete populations.

**Results:**

From the initial 5943 articles identified, 2863 duplicates were removed, followed by a further 2833 articles from title and abstract selection. Manual searching of the reference lists of the remaining 247 articles, together with use of the Google Scholar ‘cited by’ tool, yielded 205 extra articles deemed worthy of assessment. Sixty-eight studies were subsequently selected for inclusion in this study, of which 45 investigated injury only, 17 investigated illness only, and 6 investigated both injury and illness. This systematic review highlighted a number of key findings, including disparity within the literature regarding the use of various terminologies such as training load, fatigue, injury and illness. Athletes are at an increased risk of injury/illness at key stages in their training and competition, including periods of training load intensification and periods of accumulated training loads.

**Conclusions:**

Further investigation of individual athlete characteristics is required due to their impact on internal training load and, therefore, susceptibility to injury/illness.

## Key Points

**Table:** 

Athletes training load and fatigue should be monitored and modified appropriately during key stages of training and competition, such as periods of intensification of work training load, accumulated training load and changes in acute training load, otherwise there is a significant risk of injury.
Immunosuppression occurs following a rapid increase in training load. Athletes who do not return to baseline levels within the latency period (7–21 days) are at higher risk of illness during this period.
Individual characteristics such as fitness, body composition, playing level, injury history and age have a significant impact on internal training loads placed on the athlete. Longitudinal management is therefore recommended to reduce the risk of injury and illness.

## Introduction

Previous research has demonstrated that training and competition stress result in temporary decrements in physical performance and significant levels of fatigue post-competition [1–3]. These decrements are typically derived from increased muscle damage [3, 4], impairment of the immune system [1], imbalances in anabolic–catabolic homeostasis [5], alteration in mood [6, 7] and reduction in neuromuscular function (NMF) [2, 7, 8]. The resultant fatigue from these variables can take up to 5 days to return to baseline values post-competition [5], with sports that have frequent competition (i.e. often weekly in team sports) also inducing accumulative fatigue over time [9]. In addition to the significant amounts of fatigue induced by competition, many athletes experience fatigue as a result of the work required to develop the wide variety of physical qualities that contribute significantly to performance. For example, in both team and individual sports, speed, strength, power and endurance are required in addition to technical and tactical skills [10]. To achieve optimal development and performance, these physical qualities must be trained and developed, which, irrespective of the level of training loads used, may also induce further levels of fatigue [10, 11].

### Training Load, Fatigue, Injury and Illness Definitions

Training load, fatigue, injury and illness have become widely used terms within exercise science and sports such as soccer and the various rugby codes; however, there has been a lack of consistency regarding these definitions and their use. When describing load/workload throughout this paper, unless otherwise stated, load refers to training load and is defined as the stress placed on the body by the performed activity [12]. Training load comprises internal and external workload, whereby internal training load quantifies the physical loading experienced by an athlete and external training load describes the quantification of work external to the athlete [13]. Fatigue can be defined as the decrease in the pre-match/baseline psychological and physiological function of the athlete [14]. An accumulation of fatigue can result in overtraining, which has a significant negative impact on performance [15]. For example, the investigation by Johnston et al. [16] regarding the physiological responses to an intensified period of rugby league competition over a 5-day period found that cumulative fatigue appeared to compromise high-intensity running, maximal accelerations and defensive performance in the final game. This suggests that when athletes do not receive adequate time to recover between training and competition, fatigue will accumulate, compromise key aspects of performance and result in an increased risk of injury and illness to the athlete [1, 15–17]. The definition of injury has recently been realigned to the notion of impairment used by the World Health Organization [18, 19]. As a result injury can be categorised into three domains: clinical examination reports, athlete self-reports and sports performance, according to the Injury Definitions Concept Framework (IDCF) [18, 19].

### Monitoring Tools

Due to the highly complex nature of fatigue [9, 20], as well as individualised responses to similar training loads [21, 22], it is important to monitor global athlete fatigue levels (i.e. mental, physical and emotional) in response to prescribed training loads in order to minimise injury and illness [23]. Given the link between training load and injury incidence is now established, measures aimed at controlling and reducing the risk factors for the development of a sports injury are critical to primary, secondary and tertiary injury prevention [149]. Monitoring tools are used extensively in elite sport as valid indicators of recovery status of the athlete [17] and to inform support staff making decisions regarding the balance between prescribing training and recovery/rest so that performance is optimised and injury/illness minimised. Various aspects of global training load and fatigue can be measured that impact the day-to-day readiness of the athlete [17], with a range of subjective and objective measures adopted to monitor both load (e.g. training volume/duration/exposure, number of skill repetitions, rating of perceived exertion [RPE], session RPE [sRPE], global positioning systems [GPS]) and fatigue (e.g. perceptual wellness scales, neuromuscular fatigue, biochemical markers, immunological markers and sleep quantity/quality) [17].

### The Relationship Between Training Load and Fatigue Markers and Injury and Illness

The majority of training load/fatigue monitoring research has focused on acute responses to measure recovery of performance variables and the acceleration of this process through the implementation of recovery modalities [8, 24, 25]. In contrast, fewer attempts have been made to monitor acute and/or cumulative load and fatigue variables longitudinally to determine the association with injury/illness. Longitudinal monitoring refers to the investigation of how change or accumulation in training load/fatigue is associated with injury/illness over time. The use of long-term monitoring allows for the measurement of training load and fatigue variables to identify any injury/illness trends in order to provide practitioners with objective data for planning training over multiple blocks, rather than relying solely on anecdotal evidence, with the aim of reducing overtraining and injury/illness [17, 26]. Any subsequent reduction in injury and illness is likely to have a significant impact on team performance due to the large percentages of athletes from training squads (approximately 25 %) in team sports injured at any one time [27], and the association between the number of injuries and matches won [28, 29]. Although recent reviews have provided a summary of the methods available to monitor athlete load and fatigue [17], the relationship between training load in throwing-dominant sports [144], training load and injury, illness and soreness [13], and the relationship between workloads, physical performance, injury and illness in adolescent male football players [150], they have not detailed or critiqued the specific relationship between longitudinal training load, fatigue markers, and subsequent injury and illness. Additionally, previous reviews have adopted strict inclusion criteria, leading to lower numbers of studies included for consideration.

### Objectives

The objective of this study was to perform a systematic review and evaluate the association between longitudinally monitored training load, markers of fatigue, and injury/illness in sporting populations. In doing so, this review gives recommendations regarding appropriate variables to measure training load, and suggestions for further studies investigating longitudinal monitoring and fatigue markers and their relationship with injury and illness.

## Methods

### Literature Search Methodology

A Cochrane Collaboration [30] review methodology (literature search, assessment of study quality, data collection of study characteristics, analysis and interpretation of results, and recommendations for practice and further research) was used to identify relationships between long-term training load, fatigue markers, injury and illness.

### Search Parameters and Criteria

**Fig. 1:**
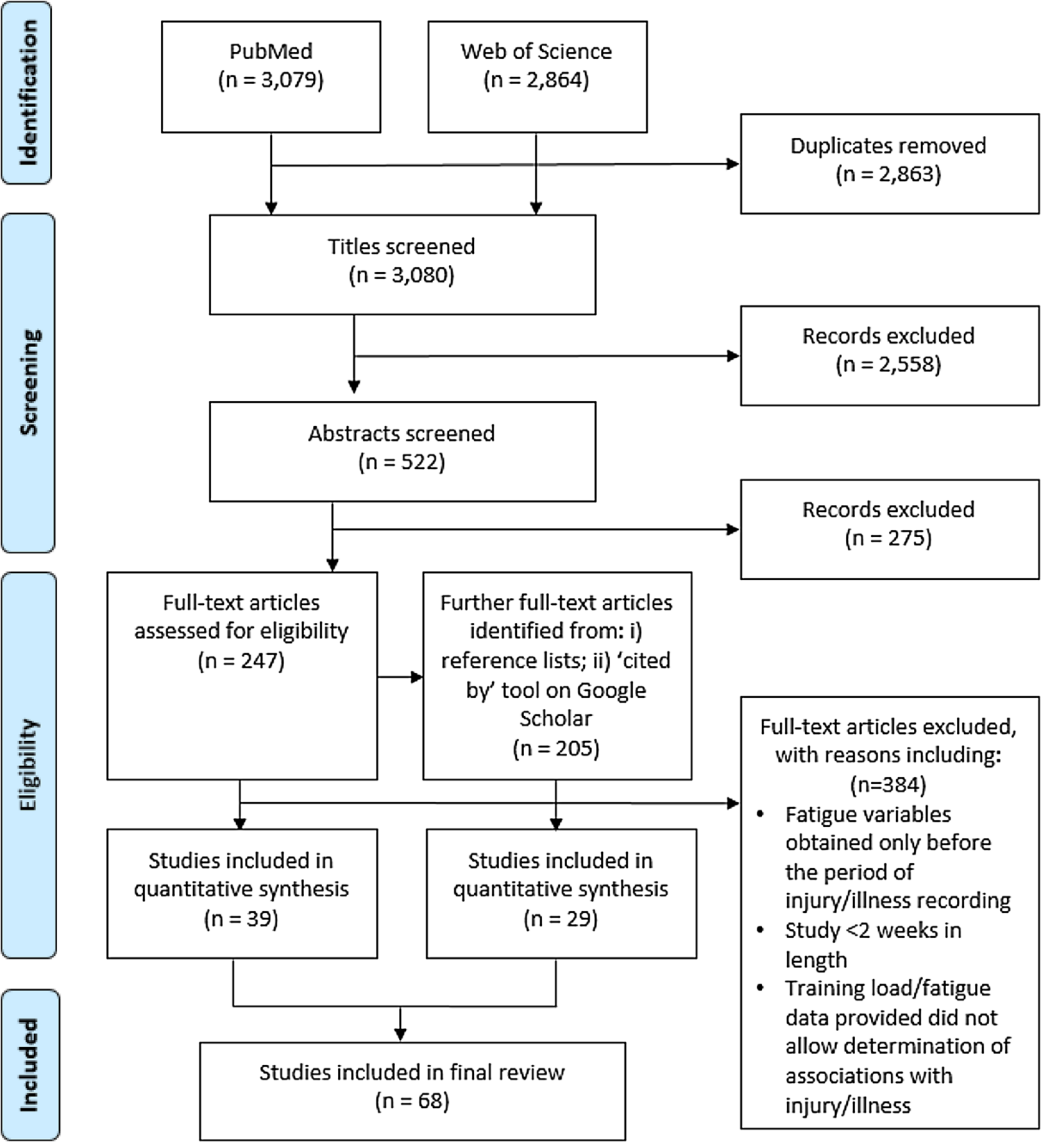
Flow of information through the systematic review process

We searched the Web of Science and PubMed online databases until August 2015 using combinations of the following terms linked with the Boolean operators ‘AND’ and ‘OR’: ‘athlete’, ‘distance’, ‘fatigue’, ‘illness’, ‘injury*’, ‘match’, ‘monitor*’, ‘monitoring’, ‘neuromuscular’, ‘performance’, ‘training’, and ‘wellness’. Articles were first selected by title content, then abstract content, and then by full article content. Manual searches were then conducted from the reference lists of the remaining articles that were selected for the ‘full article content’ stage, using the Google Scholar ‘cited by’ tool and article reference lists. Exclusion criteria included studies that were (i) unavailable in English; (ii) review papers; (iii) purely epidemiological; (iv) studying non-athlete, chronically sick and/or already injured/ill populations; (v) study length <2 weeks; and (vi) acute studies not investigating how change or accumulation in load/fatigue associates with injury/illness over time (Fig. 1 shows the flow of information through the systematic review process). After an initial 5943 articles were identified through online database searching, 2863 were discarded due to duplication, 2558 were discarded due to title content, and 275 were discarded due to abstract content. Subsequent manual searching yielded 205 additional articles that were also assessed for inclusion. Lastly, 384 articles were discarded upon assessment of their full-text content, leaving 68 studies for inclusion in the final review (injury, *n* *=* 45; illness, *n* *=* 17; injury and illness, *n* *=* 6).

### Assessment of Study Quality

As noted in previous systematic reviews [31], the usual method of quality evaluation comprises tools such as the Delphi [32] or PEDro (Physiotherapy Evidence Database) [33] scales whose criteria are often not relevant for specific review study types, including this current review article. For example, similar to Hume et al. [31], 5 of the 11 PEDro scale criteria were not included by any study in this review, including concealed allocation, subject blinding, therapist blinding, assessor blinding and intention-to-treat analysis. Therefore, to reduce the risk of bias, and given the unsuitability of scales such as Delphi and PEDro to assess the literature in this review, two authors independently evaluated each included article using a 9-item custom methodological quality assessment scale with scores ranging from 0 to 2 (total score out of 18). The nine items included (1) study design (0 = retrospective, 1 = prospective cohort, 2 = experimental e.g. intervention or case/control); (2) injury and/or illness inclusion (1 = one of either, 2 = both); (3) injury/illness definition (0 = not stated, 1 = no distinction between performance, self-reported or clinical, 2 = clearly defining if injury was sports performance, self-reported or clinical examination; (4) sporting level (0 = less than sub-elite, 1 = sub-elite, 2 = elite); (5) fatigue and/or load inclusion (1 = one of either, 2 = both); (6) number of fatigue and/or load variables (0 = 1, 1 = 2–3, 2 = more than 3); (7) statistics used (0 = subjective/visual analysis or no direct comparative analysis for fatigue/load and injury/illness associations; 1 = objective statistics for fatigue/load and injury/illness associations, 2 = objective statistics with: (i) adjustments for fatigue/load interactions, or (ii) quantification of injury/illness prediction success); (8) study length (0 = less than 6 weeks, 1 = 6 weeks to 1 year, 2 = more than 1 year); and (9) fatigue and/or load monitoring frequency (0 = less than monthly, 1 = weekly to monthly, 2 = more than weekly). Item 4 (sporting level) definitions were as follows: less than sub-elite—unpaid novices or recreational athletes, e.g. first-time runner [35] or amateur rugby league player who trains once or twice a week and plays weekly matches [36]; sub-elite—experienced athlete who trains regularly with a performance focus, e.g. lower-league soccer player who trains two to three times a week [37]; elite–athletes competing and/or training at national or international level. Item 6 (load/fatigue variables) refers to the number of a particular kind of variable. For example, three immunological markers and five perceptual wellness factors included in a study would be registered as two variables, not eight. A positive approach was taken regarding items 6 and 9, i.e. the variable that scored the greatest on the item scale was included as the final score. For example, if one variable was monitored twice a week and one was measured monthly, the final score for item 9 would be 2. The mean ± standard deviation (SD) study quality score was 11 ± 2 (range 7–15).

### Data Extraction and Analysis

For each article, the year of publication, quality score, sex, sporting level, sample size, injury/illness definition and type, fatigue/load variables, and a summary of findings were extracted and are included in Tables 1, 2, 3 and 4. Only the fatigue/load variables that were associated with injury/illness in each study were included. As much data as possible were included for the summary of findings; however, if large amounts of data were reported in an article then only significant/clear results were used. The magnitude of effects were reported in the following descending order of priority: (i) objective statistics such as risk ratios or mean differences; and (ii) visual trends or descriptive results of data with no statistical test. The probability of effects in the summary findings were reported in the following descending order of priority: (i) exact *p* values; (ii) significance levels (e.g. *p* < 0.05, *p* < 0.01); and (iii) 95 % confidence intervals (CIs). To preserve table space, differences in group means was reported rather than the raw values for each group comparison e.g. +15 rather than 25 versus 10. Although three studies initially provided no direct statistics to assess load/fatigue–injury associations [36, 38, 39], raw group load/fatigue–injury data were analysed using Pearson correlations. A priori level of evidence was evaluated using the van Tulder et al. method [142]. Levels of evidence were defined as strong (consistent findings among multiple high-quality randomised controlled trials [RCTs]), moderate (consistent findings among multiple low-quality RCTs and/or non-RCTs, clinical controlled trials [CCTs], and or one high-quality RCT), limited (one low-quality RCT and/or CCT), conflicting (inconsistent findings among multiple trial RCTs and/or CCTs) and no evidence from trials (no RCTs or CCTs) [142]. The van Tulder et al. [142] method is an accepted method of measuring the strength of evidence [13, 142]. The Oxford Centre of Evidence-Based Medicine Levels of Evidence [151] was utilised to determine the hierarchical level of evidence, whereby the highest level of evidence pertained to a systematic review of RCTs, and the lowest level of evidence pertained to expert opinion without critical appraisal or based on physiology, bench research or ‘first principles’ [13, 151]. The levels of evidence of each study are presented in Tables 1, 2, 3 and 4.

**Table 1:** Summary of findings for studies investigating training load associations with injury

References	Quality score/18	Study design, hierarchical level of evidence	Sex/sport/level (*n*)	Injury definition^a^/type	Load measures	Summary of findings
Anderson et al. [107]	12	Prospective cohort, 2b	Female/basketball/elite (12)	Time-loss/all injury	sRPE (training load, monotony and strain)	Pearson correlations with injury: training load, *r* = 0.10 (NS); strain, *r* = 0.68***; monotony, *r* = 0.67***
Arnason et al. [40]	12	Prospective cohort, 2b	Male/soccer/elite (306)	Time-loss/all injury	Training exposure	Injured group vs. non-injured, ORs: (*p* value) match exposure (min), >1 SD below mean 0.18 (<0.001); >1 SD above mean 0.61 (0.09); training exposure (min), >1 SD below mean 0.51 (0.07); >1 SD above mean 0.34 (0.01)
Bengsston et al. [53]	12	Prospective cohort, 2b	Male/soccer/elite (27 teams)	Time-loss/muscle and ligament injury	Days between matches and number of matches	RRs, <4 days between matches vs. >6 days recovery (*p* value): all injury, league 1.1 (0.045), Europa League 0.7 (0.064); muscle injury, league 1.3 (<0.001), Europa League 0.5 (0.055); ligament injury, other cup 1.8 (0.041); all competition, hamstring injury 1.3 (0.011), quadriceps injury 1.8 (0.006) Linear regression, one match/month change and injury incidence/1000 h: same match sequence, muscle injury 1.6 (0.012); subsequent match sequence, total injury 2.0 (0.056)
Brink et al. [71]	13	Prospective cohort, 2b	Male/soccer/elite (53)	Combined/all injury	Training and match duration, and load (sRPE) [load, monotony and strain]	Injured group vs. non-injured, ORs (*p* value): traumatic injury, physical stress, duration 1.14*, load 1.01*, monotony 2.59*, strain 1.01* Overuse injury, physical stress, duration 1.1 (NS), load 1.0 (NS), monotony 0.8 (NS), strain 1.0 (NS)
Brooks et al. [27]	12	Prospective cohort, 2b	Male/rugby union/elite (502)	Time-loss/all injury	Training exposure	Training injury: average number and days lost per week significantly higher when total weekly training >9.1 h vs. <9.1 h Match injury: average severity and days lost per week significantly higher when total weekly training >9.1 h vs. 6.3–9.1 h
Buist et al. [35]	10	Prospective cohort, 2b	Mixed/runners/novice (532)	Time-loss/all injury (running related)	Training exposure	Graded (intervention) vs. standard (control) training programme: weekly increase in running minutes +13.2 % (NS); OR for injury (95 % CI) 0.8 (0.6–1.3) [NS]
Carling et al. [38]^b^	10	Prospective cohort, 2b	Male/soccer/elite (1 team)	Time-loss/all injury	Match distance/min (total and >5.3 m/s)	Average m/min/match for each season and injury, Pearson correlation (*p* value): total m/min, severity, days *r* = 0.92 (0.025), number of matches *r* = 0.86 (0.06); >5.3 m/s m/min, muscle strain *r* = −0.91 (0.03)
Carling et al. [62]	11	Prospective cohort, 2b	Male/soccer/elite (19)	Time-loss/all injury	Days between matches	Congested match period vs. less congested match periods: injury incidence +0.5/1000 h (0.940), severity −5.9 (0.043)
Colby et al. [86]	12	Prospective cohort, 2b	Male/AF/elite (46)	Time-loss/intrinsic	Training distance, velocity and acceleration (total distance, sprint distance, V1 distance, velocity load, RVC; GPS)	Injury risk, ORs (*p* value), preseason: cumulative load, 3-week velocity load (6737–8046 vs. <6737 AU) 0.24 (0.04); 3-week sprint distance (846–1453 vs. < 864 m) 0.23 (0.05); 3-week total distance (73,721–86,662 vs. 73,721 m) 5.49 (0.01) Absolute change (±), force load (>556 vs. less than −13 AU) 0.10 (0.05); RVC load (0.1–9.4 vs. < 0.10 AU) 0.04 (0.006) Inseason: cumulative load, 3-week force load (>5397 vs. <4561 AU) 2.53 (0.03); 4-week RVC load (>102 vs. <84 AU) 2.24 (0.04); 2-week V1 distance (10,321–12,867 vs. 10,321 m) 0.41 (0.01), (>12,867 vs. 10,321 m) 0.28 (0.006); 2-week total distance (m). Absolute change (±), total distance (−549 to 6955 vs. −549 m) 0.49 (0.04), (>6955 vs. −549 m) 0.48 (0.08)
Cross et al. [73]	11	Prospective cohort, 2b	Male/rugby union/elite (173)	Time-loss/all injury	Training load (sRPE)	Injury risk, OR (95 % CI) 1-week +1245 AU 1.7 (1.1–2.7), 1-week change +1069 AU 1.6 (1.0–2.5); 4-week load (all vs. <3684 AU), 5932–8591 AU 0.6 (0.2–1.4), >8651 AU 1.4 (1.0–2.0)
Dellal et al. [63]	11	Prospective cohort, 2b	Male/soccer/elite (16)	Time-loss/all injury	Days between matches	Injury incidence/1000 h, congested vs. non-congested match periods: overall −1.2 (NS), match +24.7***, training −10***
Dennis et al. [56]	12	Prospective cohort, 2b	Male/cricket (fast bowlers)/elite (90)	Time-loss/gradual onset	Training load (days between matches and number of deliveries)	Injury rate, RRs (95 % CI) balls bowled per week (vs. 123–188 balls), <123 balls 1.4 (1.0–2.0), >188 balls 1.4 (0.9–1.6) Days between bowling sessions (all vs. 3–3.99 days) <2 days 2.4 (1.6–3.5); 2–2.99 days 1.4 (0.9–2.2); 4–4.99 days 1.3 (0.7–2.3); >5 days 1.8 (1.1–2.9)
Duckham et al. [42]	7	Prospective cohort, 2b	Female/running/mixed (70)	Combined/stress fracture	Training exposure	Training exposure (h/week) in non-stress fracture group vs. case one −3, case two +7
Dvorak et al. [43]	8	Retrospective cohort, 2b	Male/soccer/mixed (264)	Combined/all injury	Training exposure	Injured vs. uninjured players: games played previous season—+0.4 (NS); total training h/week in previous preparation period +2.6*; total training h/week in previous competition period +1.5*
Ekstrand et al. [44]	11	Prospective cohort, 2b	Male/soccer/elite (266)	Time-loss/all injury	Training exposure	World Cup vs. non-World-Cup players, mean difference: exposure (h/player), total +41***, training +20 (NS), matches +21*** Injury incidence (injuries/1000 h), total −1.6 (NS), training −2.3***, matches −3.6 (NS)
Fünten et al. [45]	10	Prospective cohort, 2b	Male/soccer/elite (188)	Time-loss/all injury	Training exposure	Mean difference, 2009–2010 (3.5 week winter break) vs. 2008–2009 season (6.5 week winter break) post-winter break: exposure (h), total −18.4 (<0.001), training −16.7 (<0.001), match −1.6 (0.15) Injury RRs, 2009–2010 vs. 2008–2009 (*p* value): all, knee ligament 1.9 (0.09); training, traumatic 1.5 (0.07), minimal 1.5 (0.02), severe 1.8 (0.06), sprain/joint 1.8 (0.07), knee ligament 3.1 (0.05); match, moderate 0.6 (0.09)
Gabbett and Domrow [106]^b^	11	Prospective cohort, 2b	Male/rugby league/recreational (68)	Combined/all injury	sRPE (training and match load)	Monthly load (sRPE) and injury rate (per 1000 h) relationships, Pearson correlations (*p* value): training load *r* = 0.40 (0.28), match load *r* = 0.35 (0.44) Significantly (*p* < 0.05) lower training loads and higher match loads corresponded with periods of highest injury rates
Gabbett et al. [64]	13	Non-RCT, 2b	Male/rugby league/elite (91)	Time-loss/non-contact soft tissue lower body	sRPE (training load)	Training load [sRPE] (95 % CI) and injury prevalence (%), when actual loads exceeded planned: preseason, 4341 (4082–4600) AU and 72 (63–81) %; early competition, 2945 (2797–3094) AU and 75 (66–84) %; late competition, 3395 (3297–3493) AU and 57 (47–67) % Training load range (sRPE) for 50–80 % likelihood of injury: preseason 3000–5000 AU, late competition 1700–3000 AU Accuracy of model for predicting injury (95 % CI) sensitivity 87.1 (80.5–91.7) %; specificity 98.8 (98.1–99.2) %; likelihood ratio positive 70.0 (45.1–108.8); likelihood ratio negative 0.1 (0.1–0.2)
Gabbett [77]	9	Prospective cohort, 2b	Male/rugby league/sub-elite (79)	Combined/all injury	sRPE (training and match load)	Injury incidence, Pearson correlations: training injury, intensity (RPE) *r* = 0.83*; duration (min) *r* = 0.79*; load (sRPE) *r* = 0.86* Match injury, intensity (RPE) *r* = 0.74*; duration (min) *r* = 0.86*; load (sRPE) *r* = 0.86*
Gabbett [78]	11	Non-RCT, 2b	Male/rugby league/sub-elite (220)	Sports performance and time-loss/all injury including mechanism	sRPE (training load)	Differences between 2001 and 2002/2003 preseasons (*p*-values): training intensity (RPE), 2003 vs. 2001 −0.3 2011***; training load (sRPE) vs. 2001, 2002 season −65 AU***, 2003 season −28 AU** Injury incidence (injury/1000 h) vs. 2001, all injury 2002 −62.3***, 2003 −78.3***, time-loss injury 2002 −3.3**, 2003 −14.4**
Gabbett and Domrow [79]	11	Prospective cohort, 2b	Male/rugby league/sub-elite (183)	Time-loss/all injury	sRPE (training load)	Individual level, one unit change in log of training load/week and injury risk, OR (*p* value): preseason 2.12 (0.01); early competition 2.85 (0.01); late competition 1.50 (0.04) Group level, influence of one unit change in training load/week (AU) on change in injury incidence/1000 h (*p* value): pre-season +0.35 (0.01); early competition −0.08 (0.53); late competition +0.02 (0.84)
Gabbett and Jenkins [80]	14	Prospective cohort, 2b	Male/rugby league/elite (79)	Combined/non-contact and contact and activity type	sRPE (training load)	Relationships between total, field and strength training load (sRPE) and injury, Pearson correlations: total injury, total *r* = 0.82**; field *r* = 0.67*; strength *r* = 0.81** Field injury, total *r* = 0.86**; field *r* = 0.68*; strength *r* = 0.87**; non-contact injury, total *r* = 0.82**; field *r* = 0.65*; strength *r* = 0.82** Contact injury, total *r* = 0.80**, field *r* = 0.63*, strength *r* = 0.75**; strength injury, total *r* = 0.59 (NS); field *r* = 0.43 (NS); strength *r* = 0.63*
Gabbett and Ullah [34]	11	Prospective cohort, 2b	Male/rugby league/elite (34)	Sports performance and time-loss/non-contact soft tissue lower body	Training distance (m for various velocity thresholds and m/min; GPS)	Relative risk of injury for thresholds of training load [m/session] (threshold load value): very low intensity (>542 m), time-loss injury 0.4*; low intensity (>2342 m), time-loss injury 0.5*; very high intensity (>9 m), sports performance injury 2.7*; mild acceleration (>186 m), sports performance injury 0.2**; moderate acceleration (>217 m), sports performance injury 0.3**, time-loss injury 0.4*; maximum acceleration (>143 m), sports performance injury 0.4*, time-loss injury 0.5*
Gabbett et al. [64]	10	Prospective cohort, 2b	Male/rugby league/elite (30)	Combined/collision injury	Number and intensity (g experienced; GPS accelerometer) of collisions and days between matches	Number of training collisions and training collision injury rate both significantly (*p* < 0.05) higher in 10-day recovery cycles between matches than <10-day recovery cycles
Gabbett et al. [65]	11	Prospective cohort, 2b	Male/rugby league/elite (51)	Time-loss/collision injury	Number of collisions (coded from video footage) and days between matches	Match collisions significantly (*p* < 0.05) greater in wide-running position vs. all other positions, but significantly lower collision injury rate; match collision injury rate/10,000 collisions significantly (*p* < 0.05) higher in 8-day recovery cycles between matches than >/<8-day recovery cycles
Hägglund et al. [46]	10	Prospective cohort, 2b	Male/soccer/sub-elite (26)	Time-loss/all injury	Training and match number and exposure	2001 vs. 1982 seasons for 15 best players/team (*p* values): training sessions (player/year) +76 (<0.001); matches (player/year) −8 (<0.001); training exposure (h/player) +97 (<0.001), match exposure (h/player) −12 (<0.001) Injury incidence/1000 h, training +0.6 (0.63), matches +5.3 (0.45), slight −0.8 (0.53), minor +0.1 (0.86), moderate +0.5 (0.30), major −0.1 (0.65)
Hägglund et al. [47]	11	Prospective cohort, 2b	Male/soccer/elite (188)	Time-loss/all injury including mechanism	Training and match number and exposure	Swedish vs. Danish 2001 spring domestic season (*p* values): training sessions (player/year) +34 (<0.001); matches (player/year) +1 (0.52); training exposure (h/player) +48 (<0.001); match exposure (h/player) −1 (0.23) Injury incidence, player/season, training −0.4 (0.001), matches −0.2 (0.29); 1000/h, training −5.8 (<0.01), matches −2.0 (0.59), slight −3.1 (0.088), minor −1.5 (0.014), moderate −0.5 (0.15), major −1.1 (0.002)
Hulin et al. [57]	13	Retrospective cohort, 2b	Male/cricket (fast bowlers)/elite (28)	Time-loss/non-contact	sRPE (training load) and balls bowled/week	Relationship between increased training load and injury risk, RRs (*p* value): external load (balls bowled/week), acute (1-week), same week decreased injury (0.0001); chronic (4-week average), same week decreased injury (0.002), subsequent week decreased injury (0.02) Acute:chronic load ratio >100 % vs. <100 %, subsequent week injury, external load 2.1 (0.01); internal load 2.2 (0.009) Acute:chronic load ratio, RRs (*p* values), external load 200 vs. 50–99 % 3.3 (0.03), <49 % 2.9 (0.04); internal load 200 vs. 50–99 % 4.5 (0.009), <49 % 3.4 (0.03)
Killen et al. [81]	11	Prospective cohort, 2b	Male/rugby league/elite (36)	Combined/all injury	sRPE (training load, monotony, strain)	Weekly load/fatigue–injury relationships, Pearson correlations (*p* value): load (sRPE), *r* = 0.02 (0.94); strain, *r* = 0.09 (0.78); monotony, *r* = 0.32 (0.28)
Main et al. [50]	14	Prospective cohort, 2b	Mixed/triathlon/sub-elite (30)	Combined/all injury	Training exposure and sessions/week and perceived effort and intensity (1–5 scale)	Linear mixed model associations with signs and symptoms of injury and illness: total number of sessions/week***, swim sessions/week*, cycle sessions/week**, running sessions/week***
Mallo and Dellal [55]	13	Prospective cohort, 2b	Male/soccer/elite (35)	Time-loss/ligament sprains and muscle strains	Training heart rate, number of sessions and session frequency	Ligament sprains higher in first two training stages*; muscle strains higher in final training stage (*p* = 0.051) Injury incidence relationships with stage training load, Pearson correlation: heart rate *r* = 0.72*; training frequency *r* = −0.17 (NS); number of sessions *r* = −0.20 (NS)
Murray et al. [66]	11	Prospective cohort, 2b	Male/rugby league/elite (43)	Time-loss/all injury	Days between matches	Injury incidence/1000 h for short (5–6), medium (7–8) and long (9–10) days between matches: no differences for all injuries between different cycles; significantly fewer buttock, thigh and muscular injuries after short cycles**; adjustable highest injury incidence after short cycles and hit-up forwards and outside backs after long cycles**
Nielsen et al. [87]	11	Prospective cohort, 2b	Mixed/running/novice (60)	Time-loss/all injury (running related)	Training distance (GPS)	Mean differences (*p* value): injured increase in weekly training load vs. non-injured +9.5 % (0.07); increase in training load week before injury vs. all other weeks +86 % (0.03)
Orchard et al. [60]	12	Retrospective cohort, 2b	Male/cricket (fast bowlers)/elite (129)	Time-loss/non-contact or gradual onset bowling injury	Training load (overs bowled)	5.4 (18.8 %) more overs bowled/match in players injured in the next 28 days vs. non-injured RRs (95 % CI) injury risk for >50 overs bowled/match in the following: 14 days 1.8 (1.0–3.3); 21 days 1.8 (1.1–3.0); 28 days 1.6 (1.0–2.6)
Orchard et al. [58]	12	Prospective cohort, 2b	Male/cricket (fast bowlers)/elite (235)	Time-loss/non-contact or gradual onset bowling injury	Training load (overs bowled)	RRs (95 % CI) for injury: overs bowled in time period and injury risk for following 28 days: 5 days >50 overs 1.5 (1.0–2.3), 17 days >100 overs 1.8 (0.9–3.5)
Orchard et al. [59]	12	Prospective cohort, 2b	Male/cricket (fast bowlers)/elite (235)	Time-loss/non-contact or gradual onset bowling injury	Training load (overs bowled)	Tendon injury in 21 days, RRs (*p* value): match >50 overs 3.7 (0.001), career >12,000 overs 2.4 (0.000), previous season >400 overs 2.0 (0.000), 3 previous months >150 overs 0.3 (0.000), career >3000 overs 0.2 (0.000); bone-stress injury in 28 days, 3 previous months >150 overs 2.1 (0.000); muscle injury in 28 days, previous season >400 overs 0.7 (0.020); joint injury in 28 days, previous season >450 overs 2.0 (0.015)
Owen et al. [67]	13	Prospective cohort, 2b	Male/soccer/elite (23)	Time-loss/all injury	Training heart rate (T-HI and T-VHI)	Injury and heart rate relationships (*p* value): Pearson correlations, T-HI, training *r* = 0.57 (0.005), match *r* = 0.09 (0.69), traumatic 0.42 (0.04), severity 0.51 (0.01); T-VHI, training *r* = 0.57 (0.005), match *r* = 0.19 (0.38), traumatic 0.44 (0.03), severity 0.47 (0.02) Forwards stepwise linear regression, T-HI and T-HVI *r* ^2^ = 0.28 (0.014); OR (*p* value): T-HI, match injury 1.9 (0.02) Less T-HI (*p* = 0.06) and T-VHI (*p* = 0.04) in the month before an injury did not occur vs. an injury occurring
Piggott et al. [68]	13	Prospective cohort, 2b	Male/AF/elite (16)	Time-loss/all injury	sRPE (training load, monotony and strain), mins >80 % Maximum heart rate, training distance (total and > 3.3 m/s; GPS)	Injury incidence relationships, Pearson correlations (*p* values): load (NS), monotony *r* = 0.25 (NS), strain *r* = 0.07 (NS), distance *r* = −0.52 (0.05), distance >3.3 m/s (NS), time >80 % maximum heart rate (NS) Percentage of injury explained by previous spike: load, 40 %; strain, 40 %; monotony, 20 %
Putlur et al. [84]	13	Prospective cohort, 2b	Female/soccer/sub-elite (14 plus 14 recreational controls)	Time-loss/all injury	sRPE (training load, monotony and strain)	Mean training load, monotony and strain and injury frequency greater in soccer vs. control group
Rogalski et al. [85]	12	Prospective cohort, 2b	Male/AF/elite (46)	Time-loss/all injury	sRPE (training and match load)	Injury, ORs (*p* value): training load (sRPE), 1-week load all vs. <1250 AU, 1250–1750 AU 1.95 (0.06), 1750–2250 AU 2.54 (0.007), >2250 AU 3.38 (0.001); 2-week load, all vs. <2000 AU, 2000 to <3000 AU 2.93 (0.14), 3000–4000 AU 4.03 (0.05), >4000 AU 4.74 (0.03) Previous to current week change, all vs. 250 AU, 250–750 AU 1.34 (0.15); 750–1250 AU 0.89 (0.68); >1250 AU 2.58 (0.002)
Saw et al. [61]	10	Prospective cohort, 2b	Male/cricket/elite (28)	Combined/throwing associated injuries	Training load (number of throws in training and matches)	Mean differences (*p* value): injured vs. non-injured, throws/day +12.5 (0.06), throws/week +49.7 (0.004); week before injury vs. all other weeks prior to injury, throws/week +38.9 (0.0001), throwing days/week +1.9 (0.04), rest days vs. throwing days −2.2 (0.0004)
van Mechelen et al. [51]	9	Prospective cohort, 2b	Mixed/mixed/recreational (139)	Time-loss/all injury	Training exposure	Injury OR (95 % CI) for total sporting time above median (4050 h) 6.9*
Veugelers et al. [70]	11	Prospective cohort, 2b	Male/AF/elite (45)	Time-loss/non-contact soft tissue injury	RPE and sRPE (all training and field training load)	High vs. low training load (above and below median), ORs for injury (*p* values): all training, sRPE, 1 week 0.20 (0.04), RPE, 1 week 0.20 (0.04), 2 weeks 0.23 (0.06)
Viljoen et al. [52]	9	Prospective cohort, 2b	Male/rugby/elite (38)	Combined/all injury	Training load (overs bowled)	In-season, training h/match, 3-year decrease; injury rates, 3-year decrease Pre-season, training exposure, 3-year decrease*; injury rate, 3-year increase**

*AF* Australian Football, *AU* arbitrary units, *CI* confidence interval, *g* gravitational acceleration constant, *GPS* global positioning system, *NS* non-significant, *OR* odds ratio, *RCT* randomised controlled trial, *RPE* rate of perceived exertion, *RR* risk ratio, *RVC* relative velocity change, *sRPE* session rate of perceived exertion, *T-HI* time spent at high intensity, 85–89 % of maximum heart rate, *T-VHI* time spent at very high intensity, ≥90 % of maximum heart rate, *V1* aerobic threshold speed, *2b* ‘Individual cohort study’determined by the Oxford Centre of Evidence-Based Medicine [151]

* Indicates *p* significant to 0.05 level

** Indicates *p* significant to 0.01 level

*** Indicates *p* significant to 0.001 level

^a^Combined refers to clinical, sports performance and self-reported injuries being included together in analyses, with no distinction between them

^b^Statistics derived from the raw data provided

**Table 2:** Summary of findings for studies investigating fatigue associations with injury

References	Quality score/18	Study design, hierarchical level of evidence	Sex/sport/level (*n*)	Injury definition^a^/type	Fatigue measures	Summary of findings
Brink et al. [71]	13	Prospective cohort, 2b	Male/soccer/elite (53)	Combined/all injury	REST-Q	Injured group vs. non-injured, ORs (*p* value): traumatic injury, psychological stress, fitness/injury 1.3, overuse injury, psychological stress, fitness/injury 1.5
Dennis et al. [97]	11	Prospective cohort, 2b	Male/AF/elite (22)	Time-loss/all injury	Sleep exposure and efficiency (actigraphy)	Injury week vs. two weeks before injury, two-way ANOVA (*p* value): sleep duration (min) −23 (0.47); sleep efficiency (%) −3 (0.56); sleep duration and efficiency interaction (0.62)
Gabbett and Domrow [106]^b^	11	Prospective cohort, 2b	Male/rugby league/recreational (68)	Combined/all injury	Anthropometry (sum of skinfolds, height, body mass), linear speed (40-m acceleration), lower-body power (vertical jump), agility (L run), maximal aerobic power	No clear trends for anthropometry and fitness measure changes with injury rates
Ivarsson and Johnson [37]	9	Prospective cohort, 2b	Male/soccer/sub-elite (48)	Time-loss/all injury	Hassles and Uplifts Scale	Injured group greater daily hassle pre-injury than non-injured group (*p* = 0.085)
Ivarsson et al. [93]	10	Prospective cohort, 2b	Mixed/soccer/elite (56)	Time-loss/all injury	Hassles and Uplifts Scale	Path analysis: daily hassle, direct positive effect on injury frequency***
Ivarsson et al. [94]	10	Prospective cohort, 2b	Mixed/soccer/elite (101)	Time-loss/all injury	Hassles and Uplifts Scale	Change in hassle/uplift prediction of injury incidence, latent growth-curve analysis: daily hassle +0.33**; daily uplift −1.87**
Killen et al. [81]	11	Prospective cohort, 2b	Male/rugby league/elite (36)	Combined/all injury	Perceptual wellness scores (sleep, food, energy, mood and stress; 1–10 scale)	Weekly fatigue–injury relationships, Pearson correlations (*p* value): total perceptual wellness scores *r* = 0.71 (0.08)
Kinchington et al. [95]	10	Prospective cohort, 2b	Male/AF, rugby union and rugby league/elite (182)	Time-loss/all lower-limb injury	Lower-Limb Comfort Index (36-point questionnaire)	Relationships with Lower-Limb Comfort Index and injury, Pearson correlations: poor comfort *r* = 0.88***; usual comfort 0.69***; high comfort 0.39*** Injury incidence/1000 h: poor comfort 43.5; usual comfort 14.1; high comfort 2.3
King et al. [39]^b^	7	Prospective cohort, 2b	Male/rugby league/recreational (30)	Sports performance and time-loss/all injury	REST-Q	Injury relationships, Pearson correlations (*p* value): training (sports performance injury), lack of energy *r* = −0.77 (0.04), physical complaints *r* = −0.87 (0.01), social recovery *r* = 0.69 (0.09), sleep quality *r* = 0.87 (0.01), injury *r* = −0.78 (0.04); match (time-loss injury), lack of energy *r* = −0.90 (0.005), physical complaints *r* = −0.73 (0.07), disturbed breaks *r* = −0.75 (0.05); match (sports performance and time-loss injury), lack of energy *r* = −0.72 (0.05), physical complaints *r* = −0.75 (0.07), emotional stress *r* = −0.69 (0.08)
Laux et al. [96]	11	Prospective cohort, 2b	Male/soccer/elite (22)	Time-loss/all injury	REST-Q	Injury risk month after assessment, ORs for one unit increase in REST-Q measure (*p* value): fatigue 1.7 (0.007), sleep quality 0.5 (0.010), disturbed breaks 1.8 (0.047), injury 1.8 (<0.001)
Main et al. [50]	14	Prospective cohort, 2b	Mixed/triathlon/sub-elite (30)	Combined/all injury	PSS	Linear mixed model associations with signs and symptoms of injury and illness: PSS***
Piggott et al. [68]	13	Prospective cohort, 2b	Male/AF/elite (16)	Time-loss/all injury	Salivary IgA and cortisol	Injury incidence, Pearson correlations (*p* value): week 5 cortisol *r* = 0.73*

*AF* Australian Football, *ANOVA* analysis of variance, *Ig* immunoglobulin, *OR* odds ratio, *PSS* Perceived Stress Scale, *REST-Q* Recovery-Stress Questionnaire for Athletes, *2b* ‘Individual cohort study’ determined by the Oxford Centre of Evidence-Based Medicine [151]

* Indicates *p* significant to 0.05 level

** Indicates *p* significant to 0.01 level

*** Indicates *p* significant to 0.001 level

^a^Combined refers to clinical, sports performance and self-reported injuries being included together in analyses, with no distinction between them

^b^Statistics derived from the raw data provided

**Table 3:** Summary of findings for studies investigating training load associations with illness

References	Quality score/18	Study design, hierarchical level of evidence	Sex/sport/level (*n*)	Illness definition^a^/type	Load measures	Summary of findings
Anderson et al. [107]	12	Prospective cohort, 2b	Female/basketball/elite (12)	Time-loss/all illness	sRPE (training load, monotony and strain)	Pearson correlations with illness: training load, *r* = 0.10 (NS)
Brink et al. [71]	13	Prospective cohort, 2b	Male/soccer/elite (53)	Time-loss/all illness	Training and match duration and load [sRPE] (load, monotony and strain)	Injured group vs. non-injured, ORs for illness (*p* value): physical stress, duration 1.12 (NS), load 1.00 (NS), monotony 2.52 (NS), strain 1.00 (NS)
Cunniffe et al. [54]	10	Prospective cohort, 2b	Male/rugby union/elite (31)	Combined/URI	sRPE (training load) and game number	Visual trend for reduced game time and increase training load to precede clusters of URIs
Fahlman and Engels [90]	10	Prospective cohort, 2b	Male/AmF/elite (75 plus 25 non-sporting controls)	Combined/URTI	Baecke Physical Activity Questionnaire	Football players vs. controls (*p* value): time points 2, 3, 6 and 7, higher URTI %*; all study, physical activity questionnaire, work +1 (0.78), sport +2 (0.001), leisure −1 (0.64), total +2.6 (0.003)
Ferrari et al. [74]	11	Prospective cohort, 2b	Male/road cycling/sub-elite (8 plus male college athlete controls)	Combined/URI	sRPE (training load, monotony and strain)	Training strain relationships, Pearson correlations (*p* values): WURSS score, preparatory phase *r* = 0.72 (0.03), second competitive phase *r* = 0.70 (0.05); total URTI symptoms *r* = 0.73 (0.04)
Foster [75]	11	Prospective cohort, 2b	Mixed/swimming/mixed (25)	Unknown/all illness	sRPE (training load, monotony and strain)	Percentage of illness explained by spike in individual training load thresholds: load 84 %, monotony 77 %, strain 89 % Percentage of excursions above individual thresholds that did not result in illness: load 55 %; monotony 52 %; strain 59 %
Freitas et al. [76]	11	Prospective cohort, 2b	Male/soccer/elite (11)	Combined/URI	sRPE (training load)	Higher training load in overload vs. taper phase when URI incidence was higher
Fricker et al. [69]	9	Prospective cohort, 2b	Male/running/elite (20)	Combined/all illness	Training load (distance × RPE; self-reported)	Mean training differences between week and month pre-illness and whole study average (*p* value): mileage (km), week −4 (0.65), month +7 (0.73); intensity (RPE), week 0.0 (0.87), month 0.0 (0.90); load (RPE·km), week −5 (0.82), month 32 (0.54); number of illnesses, Pearson correlations: weekly mileage, intensity and load *r* < 0.1
Gleeson et al. [88]	8	Prospective cohort, 2b	Mixed/mixed (endurance-based)/mixed (80)	Combined/all illness	MET h/week	Mean difference, ill vs. illness-free athletes (*p* value): training load (h/week) +2.3 (0.05)
Hausswirth et al. [48]	11	Prospective cohort, 2b	Male/triathlon/sub-elite (27)	Combined/URTI	Training exposure and heart rate	Frequency of total infection cases: functional overreaching group 67 %; acute fatigue group 22 %; control group 11 %
Mackinnon and Hooper [49]	10	Prospective cohort, 2b	Mixed/swimming/elite (24)	Combined/URTI	Self-reported training distance (swimming) and exposure (land-based)	Mean differences, URTI frequency, overtrained = 1/8 (12.5 %), well trained = 9/16 (56 %)
Main et al. [50]	14	Prospective cohort, 2b	Mixed/triathlon/sub-elite (30)	Combined/all illness	Training exposure and sessions/week and perceived effort and intensity (1–5 scale)	Linear mixed model associations with signs and symptoms of injury and illness: total number of sessions/week***, swim sessions/week*, cycle sessions/week**, running sessions/week***
Moreira et al. [82]	9	Prospective cohort, 2b	Male/basketball/elite (15)	Combined/URTI	sRPE (training load)	Mean differences: training load (sRPE) greater in week 2 vs. week 4*; number of URTIs higher in week 2 vs. weeks 1 and 4*
Moreira et al. [83]	11	Prospective cohort, 2b	Male/futsal/elite (12)	Combined/URTI	sRPE (training load)	Mean differences: training load (sRPE) greater in weeks 1 and 2 vs. weeks 3 and 4*; URTI severity greater in weeks 1 and 2 vs. week 4* URTI severity in week 4, Pearson correlation (*p* value): training load *r* = 0.87*
Mortatti et al. [102]	11	Prospective cohort, 2b	Male/soccer/elite (14)	Combined/URTI	Match RPE	Mean differences: match RPE greater in matches 4, 5, 6 and 7 vs. match 1*; URTI incidence greater before match 2 and 6 vs. match 1*
Neville et al. [91]	12	Prospective cohort, 2b	Male/yacht racing/elite (38)	Time-loss/URI	Combined exposure and intensity ranking (1–5 scale)	URI incidence, Pearson correlations: training exposure (sailing and training load) *r* = 0.002 (NS)
Piggott et al. [68] (2008)	13	Prospective cohort, 2b	Male/AF/elite (16)	Time-loss/all illness	sRPE (training load, monotony and strain), mins >80 % Maximum heart rate, training distance (total and >3.3 m/s; GPS)	Illness incidence relationships, Pearson correlations (*p* values): load (NS), monotony *r* = 0.12 (NS), strain *r* = 0.12 (NS), distance (NS), total distance >3.3 m/s (NS), time >80 % maximum heart rate (NS) Percentage of illness explained by previous spike: load, 42 %; strain, 25 %; monotony, 33 %
Putlur et al. [84]	13	Prospective cohort, 2b	Female/soccer/sub-elite (14 plus 14 recreational controls)	Time-loss/all illness	sRPE (training load, monotony and strain)	Mean training load, monotony and strain and illness frequency greater in soccer vs. control group; percentage of illness explained by previous spike in measure: increased training load 55 %, increased monotony and strain 64 %
Veugelers et al. [70]	11	Prospective cohort, 2b	Male/AF/elite (45)	Time-loss/all illness	RPE and sRPE (all training and field training load)	High vs. low training load (above and below median), ORs for illness (*p* values): all training, sRPE, 1 week 0.30 (0.07); field training, sRPE, 1 week 0.30 (0.07), 2 weeks 0.13 (0.05), RPE, 1 week 0.18 (0.03)

*AF* Australian Football, *AmF* American football, *GPS* global positioning system, *MET* metabolic equivalent, *NS* non-significant, *OR* odds ratio, *RPE* rate of perceived exertion, *sRPE* session rate of perceived exertion, *URI* upper respiratory illness, *URTI* upper respiratory tract infection, *WURSS* Wisconsin Upper Respiratory Symptoms Scale, *2b* ‘Individual cohort study’determined by the Oxford Centre of Evidence-Based Medicine [151]

* Indicates *p* significant to 0.05 level

** Indicates *p* significant to 0.01 level

*** Indicates *p* significant to 0.001 level

^a^Combined refers to clinical, sports performance and self-reported injuries being included together in analyses, with no distinction between them

**Table 4:** Summary of findings for studies investigating fatigue associations with illness

References	Quality score/18	Study design, hierarchical level of evidence	Sex/sport/level (*n*)	Illness definition^a^/type	Fatigue measures	Summary of findings
Brink et al. [71]	13	Prospective cohort, 2b	Male/soccer/elite (53)	Time-loss/all illness	REST-Q	Illness, psychological stress, emotional stress 2.27, social stress 2.07, conflicts/pressure 1.69, fatigue 1.48*, lack of energy 1.92, physical complaints 1.88, social recovery 0.66*, general well-being 0.57, sleep quality 0.58, disturbed breaks 1.51*, emotional exhaustion 1.47*, fitness/injury 1.60*, being in shape 0.56
Cunniffe et al. [54]	10	Prospective cohort, 2b	Male/rugby union/elite (31)	Combined/URI	Salivary lysozyme and IgA	Mean difference, present URI or ± 5 days from peak of symptoms vs. no URI (*p* value), relative IgA −15 % (0.08)
Fahlman and Engels [90]	10	Prospective cohort, 2b	Male/AmF/elite (75 plus 25 non-sporting controls)	Combined/URTI	Salivary IgA, protein and osmolality	Football players vs. controls (*p* value): time points 2, 3, 6 and 7, lower salivary IgA*, higher URTI %* Secretion rate of salivary IgA (μg/min) and number of colds (across all study time points), stepwise multiple regression: *r* ^2^ = 0.12–0.42; *p* = 0.000–0.003
Ferrari et al. [74]	11	Prospective cohort, 2b	Male/road cycling/sub-elite (8 plus male college athlete controls)	Combined/URI	Salivary IgA and leukocyte	No significant differences between training phases for any salivary immune function measure
Freitas et al. [76]	11	Prospective cohort, 2b	Male/soccer/elite (11)	Combined/URTI	Salivary cortisol and DALDA	URTI severity, Pearson correlation (*p* value): stress symptoms *r* = −0.70 (0.01); higher salivary cortisol in overload vs. taper phase when URTI incidence was higher
Gleeson et al. [98]	8	Prospective cohort, 2b	Mixed/swimming/elite (25)	Combined/URTI	Salivary IgA	Relationships between immune function markers (early and late training phase) and illness, Pearson correlations (*p* value): total IgA, early *r* = −0.56 (0.16), late *r* = −0.63 (0.10); IgA1, early −0.71 (0.01), late *r* = 0.28 (0.76); IgA2, early *r* = −0.42 (0.41), late *r* = 0.39 (0.56); IgA1:IgA2, early *r* = 0.45 (0.46); late *r* = 0.10 (0.98)
Gleeson et al. [99]	9	Prospective cohort, 2b	Mixed/swimming/elite (25)	Combined/URTI	Salivary and serum IgA/G/M and albumin, whole blood natural killer cell analysis	Median differences, infected vs. non-infected (*p* value): NK cell count (×10^9^ cells/L) +0.06 (0.14); pre-exercise, salivary IgA (mg/L) +27.5 (0.36), salivary IgM (mg/L) +1.2 (0.21), salivary IgG (mg/L) +3.1 (0.69), salivary albumin (mg/L) +6.4 (0.95); post-exercise, salivary IgA (mg/L) +12.0 (0.26), salivary IgM (mg/L) +0.3 (0.97), salivary IgG (mg/L) −0.4 (0.64), salivary albumin (mg/L) +8.3 (0.69)
Gleeson et al. [88]	8	Prospective cohort, 2b	Mixed/mixed (endurance-based)/mixed (80)	Combined/all illness	Blood cell counts, lymphocyte subsets, antigen-stimulated cytokine production, plasma immunoglobulins, salivary IgA	Mean difference, ill vs. illness-free athletes (*p* value): saliva flow rate (mL/min) −0.18 (0.004); salivary IgA secretion rate (mg/min) −31.0 (0.02); IgM (g/L) +0.45 (0.03); IL-2 production (pg/mL) +113 (0.06); IL-4 production (pg/mL) +3.9 (0.02); IL-6 production (pg/mL) +62 (0.09); IL-10 production (pg/mL) +4.4 (0.008); IFN-γ production (pg/mL) +14 (0.06)
Hausswirth et al. [48]	11	Prospective cohort, 2b	Male/triathlon/sub-elite (27)	Combined/URTI	POMS, sleep duration and efficiency (actigraphy)	Frequency of total infection cases: functional overreaching group 67 %; acute fatigue group 22 %; control group 11 %
Leicht et al. [100]	9	Prospective cohort, 2b	Mixed/wheelchair rugby/elite (14)	Combined/URS	Salivary IgA	Median difference in IgA secretion rate: illness vs. no illness (*p* = 0.19); illness within 2 weeks of sampling vs. no illness (NS)
Mackinnon and Hooper [49]	10	Prospective cohort, 2b	Mixed/swimming/elite (24)	Combined/URTI	Perceptual wellness (fatigue, stress, sleep disturbance, muscle soreness; 1–7 scale), plasma glutamine	Mean differences, overtrained vs. well-trained athletes (*p* value): perceptual wellness ratings, increased fatigue (0.02), decreased sleep quality (0.05), increased stress (0.04); plasma glutamine, time 2 −23 %*, time 3 −26 % (NS); URTI frequency, overtrained = 1/8 (12.5 %), well trained = 9/16 (56 %)
Main et al. [50]	14	Prospective cohort, 2b	Mixed/triathlon/sub-elite (30)	Combined/all illness	PSS	Linear mixed model associations with signs and symptoms of injury and illness: PSS***
Moreira et al. [82]	9	Prospective cohort, 2b	Male/basketball/elite (15)	Combined/URTI	DALDA and salivary cortisol	Mean differences: DALDA, more part A responses ‘worse than normal’ in week 2 vs. weeks 1, 3 and 4*, more part B responses ‘worse than normal’ in week 2 vs. week 4*; number of URTIs higher in week 2 vs. weeks 1 and 4*
Moreira et al. [83]	11	Prospective cohort, 2b	Male/futsal/elite (12)	Combined/URTI	Salivary cortisol and IgA	URTI severity in week 4, Pearson correlation (*p* value): relative week 1–4 ΔIgA*r* = −0.86*
Moreira et al. [101]	9	Prospective cohort, 2b	Male/soccer/sub-elite (34)	Combined/URTI	Salivary cortisol and IgA	Mean differences: IgA greater in training period 4* vs. training period 1; URTI symptoms lower in training periods 3–4* vs. training periods 1–2
Mortatti et al. [102]	11	Prospective cohort, 2b	Male/soccer/elite (14)	Combined/URTI	Salivary cortisol and IgA	Mean differences: decreased IgA before match 2 and 6 vs. match 1*; URTI incidence greater before match 2 and 6 vs. match 1* URTI incidence, Pearson correlations: decreases in salivary IgA, match 2 (*r* = −0.60)*, match 6 (*r* = −0.65)*
Neville et al. [91]	12	Prospective cohort, 2b	Male/yacht racing/elite (38)	Time-loss/URI	Salivary IgA	URI incidence, Pearson correlations: raw IgA *r* = 0.11 (NS), relative IgA *r* = 0.54** Mean differences: relative IgA, URI vs. no URI −28 %***; lower in URI week vs. −4, +1 and +2 URI weeks***; lower −1 URI week vs. +2 URI week***; chance (%) of getting URI given relative IgA, <40 % = 48 % (23/48), <70 % = 28 % (74/263)
Putlur et al. [84]	13	Prospective cohort, 2b	Female/soccer/sub-elite (14 plus 14 recreational controls)	Time-loss/all illness	Salivary IgA and cortisol	Percentage of illness explained by previous spike in measure: decreased IgA 82 %, decreased IgA and increased cortisol 55 %

*AmF* American football, *DALDA* Daily Analysis of Life Demands for Athletes Questionnaire, *IFN* interferon, *Ig* immunoglobulin, *IL* interleukin, *NK* natural killer, *L* litre, *NS* non-significant, *POMS* Profile of Mood States Questionnaire, *PSS* Perceived Stress Scale, *REST-Q* Recovery-Stress Questionnaire for Athletes, *URI* upper respiratory illness, *URS* upper respiratory symptoms, *URTI* upper respiratory tract infection, *2b* ‘Individual cohort study’determined by the Oxford Centre of Evidence-Based Medicine [151]

* Indicates *p* significant to 0.05 level

** Indicates *p* significant to 0.01 level

*** Indicates *p* significant to 0.001 level

^a^Combined refers to clinical, sports performance and self-reported injuries being included together in analyses, with no distinction between them

### Definitions of Key Terms

Training load, fatigue injury and illness have previously been defined (see Sect. 1.1). Latency period is defined as the period between training load and the onset of injury or illness [13]. Finally, we used the term ‘exposure’ to refer to time spent participating in a particular training/competition activity.

## Results

### Retention of Studies

Overall, 68 studies were retained for inclusion in the final review (Fig. 1), of which 45 (66 %) investigated injury only, 17 (25 %) investigated illness only, and 6 (8 %) investigated both injury and illness. In addition, 42 (61 %) articles focused on load–injury/illness relationships, 11 (16 %) focused on fatigue–injury/illness relationships only, and 15 (22 %) included both load and fatigue variables. In the 57 studies that investigated load–injury/illness relationships, many different load measures were used, including training exposure (*n* *=* 14, 24 %) [35, 40–52]; number of sessions/matches (*n* *=* 5, 8 %) [46, 47, 53–55], number of skill repetitions [e.g. number of deliveries bowled for cricketers] (*n* *=* 6, 10 %) [56–61]; days between/frequency of matches (*n* *=* 8, 14 %) [53, 55, 56, 62–66]; heart rate (*n* *=* 4, 7 %) [48, 55, 67, 68]; RPE (*n* *=* 2, 3 %) [69, 70]; sRPE (*n* *=* 21, 36 %) [26, 36, 40, 54, 57, 68, 70–84]; number/intensity of collisions (*n* *=* 2, 3 %) [64, 65]; distance [both self-reported and GPS derived] (*n* *=* 6, 10 %) [34, 49, 68, 69, 85, 86]; velocity/acceleration GPS-derived measures (*n* *=* 2, 3 %) [38, 85]; metabolic equivalents [MET] (*n* = 1, 1 %) [87]; the Baecke Physical Activity Questionnaire [88] (*n* *=* 1, 1 %) [89]; and a combined volume and intensity ranking [1–5 scale] (*n* = 1, 1 %) [90]. A number of fatigue measures were also used in the 26 studies that investigated fatigue–injury/illness relationships, including perceptual wellness scales (*n* *=* 13, 50 %) [37, 39, 48–50, 75, 80, 81, 91–95]; sleep quantity/quality (*n* *=* 6, 23 %) [39, 48, 71, 80, 95, 96]; immunological markers (*n* *=* 12, 46 %) [49, 54, 73, 82, 83, 87, 89, 90, 97–100]; and stress hormone levels (*n* *=* 6, 23 %) [75, 81–83, 100, 101].

### Definitions of Key Terms

Thirty-seven (54 %) studies defined injury/illness as ‘sports incapacity’ [102, 109 ] events (i.e. the injury/illness caused time-loss from, or an alteration in, normal training schedule), whereas 26 studies (38 %) did not distinguish between what category the injury/illness orientated from, and defined injury/illness by measures such as the ‘Wisconsin Upper Respiratory Symptom Survey’ [104] for upper respiratory illness (URI), or as any pain or disability experienced by a player during a match or training session [105] for injury. Only two studies did not clarify which type of injury/illness definition was used [42, 74].

### Statistical Analysis Methods

A range of statistical analysis methods were also used, including Pearson correlations [68], mean differences in load/fatigue between injured and non-injured groups [86], a Cox proportional regression frailty model [34], logistic regression with binomial distribution [26], linear regression [78] and multinomial regression [71], with only one study adjusting for interactions between load and fatigue measures [50]. Main et al. used linear mixed modelling to assess the interactive associations between training exposure and psychological stressors with signs and symptoms of illness in 30 sub-elite triathletes [50]. In addition, only two studies provided an indication of the success of load to predict injury. Specifically, Gabbett [26] achieved this using a sensitivity and specificity analysis, while Foster [74] reported the percentage excursions beyond their derived load thresholds that did not result in illness.

### Sporting Populations

A number of different sporting populations were represented, from recreational to elite level; namely, American Football (*n* *=* 1) [89]; Australian Football [AF] (*n* *=* 6) [68, 70, 84, 85, 94, 96]; basketball (*n* *=* 2) [81, 106]; cricket [fast bowlers] (*n* *=* 5) [56–58, 60, 61]; futsal (*n* *=* 1) [82]; soccer (*n* *=* 21) [37, 38, 40, 43–47, 53, 55, 62, 63, 67, 71, 75, 83, 92, 93, 95, 100, 101]; road cycling (*n* *=* 1) [73]; rugby league (*n* *=* 13) [26, 34, 36, 39, 64–66, 76–80, 94]; rugby union (*n* *=* 5) [41, 52, 54, 72, 94]; running (*n* *=* 4) [35, 42, 69, 86]; swimming (*n* *=* 4) [49, 74, 97, 98]; triathlon (*n* *=* 2) [48, 50]; wheelchair rugby (*n* = 1) [99]; and yacht racing (*n* = 1) [90]. Two studies used a mix of various sports [51, 87]. The majority of studies included only male participants (*n* *=* 52), with 11 studies including both males and females [35, 49, 50, 74, 86, 87, 92, 93, 97–99] and three including females only [42, 83, 106]. Three studies used intervention [35, 48, 77] and case-control study designs [83, 89, 105], with nine studies [38, 41, 45–47, 67, 75, 77, 82] investigating injury/illness severity as opposed to injury/illness incidence only.

## Discussion

The aim of this systematic review was to investigate the literature that has examined the longitudinal monitoring of training load and fatigue data, and its relationship with injury/illness in sporting populations. Although a number of common findings were identified from the 68 studies, a lack of consistency and conflicting views are clearly apparent within the literature regarding the definition of key terms, monitoring of the training load and injury and illness, and monitoring of fatigue markers and their relationship to injury and illness.

### Reporting of Terms

This review has identified conflicting levels of evidence for several key terms and their subsequent measures used to longitudinally monitor the athlete, including training load, fatigue, injury and illness. The use of multiple definitions within the literature to describe a singular term may lead to confusion and misuse at both a conceptual and practical level by leading to inadequate and inconsistent criteria for defining samples, and subsequent difficulty in comparing one study with another [77].

#### Training Load and Fatigue

Use of the terms training load and fatigue was found to have the greatest misinterpretation within this review, primarily the interchangeability of these terms within the literature. For example, a recent study by Hulin et al. [57] applying Bannister’s fitness–fatigue model [107] to training stress-balance (acute:chronic workload ratio) described fatigue as the acute training load (weekly training load total), and fitness as the chronic load (previous 4-weekly total average). Even though this new method of monitoring training load provided by Hulin et al. [57] has enabled a greater understanding of the relationship between training load and injury [108, 131], the application of fitness and fatigue terminology to represent training workload has added further confusion. This has resulted in several recent studies readdressing this issue, whereby the fitness–fatigue model (formerly training stress balance) has been replaced by the term acute:chronic workload ratio for this very reason [108, 131, 143, 145, 146, 148]. The key implication for researchers and practitioners here is that when training load and fatigue are used as terms they should be clearly defined and described as separate entities.

#### Injury and Illness

Along with the 37 studies that used time-loss injury/illness definitions, 26 studies have simply reported ‘injury’ or ‘illness’ when summarising key findings, without distinguishing between categories. Distinguishing between which category of injury/illness is an important practical consideration. For example, Brink et al. [71] noted differences between traumatic injury, overuse injury and illness associations with training load, while Orchard et al. [59] reported training load-related differences for joint, bone, tendon and muscle injuries. Standardised reporting of injury/illness incidence will further aid comparison between studies, as well as generation of any subsequent meta-analyses [111].

#### Exposure

Three terms were also used to describe the time spent participating in a particular training/competition activity; namely, duration [71], volume [41, 52] and exposure [44]. However, ‘volume’ was used as a term in only two studies and, in Brooks et al. [41], it was included in the study title and was used interchangeably with ‘exposure’ in the article text. Several studies, such as Buist et al. [35] and Main et al. [50], also used ‘exposure’ in the article text but did not include it in their titles or keywords.

#### Perceptual Wellness

It should also be noted that the term ‘perceptual wellness scales’ covers a range of inventories that attempt to assess how individuals perceive particular physical and psychological states. The measures used in the studies included in this review ranged from simple 1–5 Likert scale questionnaires for factors such as energy, sleep quality and mood [80], to more detailed and longer multi-question tools such as the Recovery-Stress Questionnaire for Athletes (REST-Q) [39] or the Daily Analysis of Life Demands for Athletes Questionnaire (DALDA) [81].

### Training Load and Injury

Monitoring of training load accounted for 33 of 68 studies in this review, with the majority from team sports (90 %), predominately soccer and rugby league, and the additional 10 % coming from three running studies [35, 42, 86] and one with a mixed sporting group [51] (Table 1). For internal training load, the most common measure was sRPE (*n* *=* 21), with exposure the most frequent (*n* *=* 15) for external load. The following section discusses the emerging moderate evidence for the relationship between training load and key stages of training and competition, which highlights where athletes were deemed to be more susceptible to increased risk of injury [26, 41, 44, 84, 85].

#### Periods of Training Load Intensification

Periods of training load intensification, such as preseason, periods of increased competition, and injured players returning to full training, were found to increase the risk of injury. For example, athletes returning for preseason are at significantly greater risk of injury, potentially from the intensification in training workload and detraining effect from the offseason [26, 84]. This may result in the athlete being unable to tolerate the external/internal training load placed on them. Gabbett [26] also reported that the likelihood of non-contact soft tissue injury was 50–80 % (95 % CI) in a rugby league preseason when weekly internal training load (sRPE) was between 3000 and 5000 AU compared with lower weekly training loads, and that increased loads during preseason elevated injury rate at a group level but not during the inseason [105]. Along with high preseason training loads, periods of training and match load intensification, such as periods of congested scheduling, were also investigated in the literature [7, 15, 16]. However, there was conflicting evidence from the six studies investigating associations between shortened recovery cycles and injury. Two studies found shortened recovery to be related to increased injury [53, 63], one study related to decreased injury [64], one study found moderate recovery cycles to have the highest injury risk [65], one study reported different findings depending on injury type [66], and one study found no significant associations [62].

#### Changes in Acute Training Load

Another facet of this review was how acute change in training load (week to week) is associated with injury risk. Piggot et al. [68] identified that if weekly internal training load was increased by more than 10 %, this explained 40 % of injury in the subsequent 7 days. The other two studies to assess acute changes in training load both found a positive linear relationship between increased acute internal training load (1245–1250 AU) relative to the previous week and injury rate in elite contact-sport athletes [72, 84]. However, in contrast, the investigation by Buist et al. [35] regarding injury incidence among novice runners following a graded training programme (running minutes increased 10.5 %/week) versus a control group (running minutes increased 23.7 %/week) found no difference between groups for running-related injury (RRI) rate (odds ratio 0.8, 95 % CI 0.6–1.3), despite a greater increase in acute weekly training minutes in the control group. This finding is in agreement with the study by Nielsen et al. [86] regarding the development of RRI in novice runners (*n* *=* 60) during a 10-week prospective study. Those who sustained an RRI showed an increase in weekly training load of 31.6 %/week when compared with a 22.1 %/week increase among healthy participants; however, this was deemed non-significant (*p* = 0.07). This lack of increase in injury with increased acute training load may be first explained by the fact that only external load has been measured, with all relationships that have been found adopting internal training load measures. Second, novice runners may be able to improve at a greater rate than experienced athletes [113] and are therefore potentially able to tolerate large relative increases in external training load due to the absolute external and internal training load level being low.

#### Accumulated Training Load

Another key stage of training and competition that was highlighted was the effect of long-term accumulated training load (chronic workload) on injury incidence. For example, Ekstrand et al. [44] compared external load (training/match exposures) and injury rates between elite soccer players who participated in the international World Cup after the domestic season (World-Cup players) and non-World-Cup players. World-Cup players had greater match exposure and total (training plus match) exposure compared with non-World-Cup players during the domestic season (46 vs. 33 matches). Ekstrand et al. [44] then found that 32 % of this high-exposure group sustained a drop in performance, with 29 % proceeding to sustain injury during the World Cup. Moreover, 60 % of players who had played more than one competition/week in the 10 weeks prior to the World Cup also sustained an injury. aus der Fünten et al. investigated the effect of a reduced winter break (6.5 weeks down to 3.5 weeks) by comparing training exposure and injury rates between the two seasons immediately before and after the change in the length of the winter break [45]. Even though the reduced winter break showed athletes having lower training and match exposures, injury rates were higher, particularly in training. These studies suggest that how coaches and support staff manage key stages of training and competition (e.g. the periodization of starting players, the length of offseason/midseason breaks) has significant implications regarding the maintenance of performance and reduction of injury. A specific example is the management of training/competition load of team-sport athletes during the domestic season, taking into account international and/or club competitions towards the latter end of that season [112].

#### Training–Injury Prevention Paradox

The results of this study support recent publications on the training–injury prevention paradox [103, 108, 131, 143, 145–148], whereby moderate relationships were identified between training loads and injury, yet there was disparity regarding the direction of findings (i.e. whether increased training load was associated with decreased or increased injury). For example, Brooks et al. [41] found that although higher external acute training volumes (<6.3 h/week vs. high > 9.1 h/week) did not necessarily increase elite rugby union match injury incidence, they were associated with increased severity of all injuries, especially during the latter part of the season and the second half of matches. Linear increases in acute internal training load (1245 AU) were associated with increased injury risk in a group of elite rugby union players [72] but decreased injury risk in 28 elite cricketers [57]. As well as linear training load–injury relationships, ‘U-shaped’ relationships (a phenomenon described in other scientific fields [114, 115]) were evident in several studies. For example, Dennis et al. [56] showed that bowling between 123 and 188 balls had lower injury risk than bowling <123/>188 balls. This U-shaped relationship may be due to low training loads failing to provide sufficient stimulus for attaining ‘acquired resistance’ to injury [56], and high training loads fatiguing athletes to the point where musculoskeletal tissue is less able to deal with the forces it encounters during activity [116, 117]. As with negative and positive linear training load–injury relationships, an inverted U-shaped relationship pattern [118, 119] was also elicited. For example, Arnason et al. [40] found moderate acute match and training exposures to have higher injury rates when compared with low and high exposures in elite soccer players. Collision injury rates were also higher in moderate-length recovery cycles (8 days) versus low (<8 days) and high (>8 days) recovery cycles in 51 elite rugby league players.

Another potential reason for the disparity between the findings of the relationship between training load and injury, such as the negative/positive linear and U/inverted-U patterns, is that the majority of studies report the magnitude of external load (e.g. distance or duration), but not the intensity. Increased external intensity (e.g, velocity of running and load lifted) and internal intensity (e.g. RPE and heart rate) will increase the stress placed on the body and therefore potentially increase injury risk [116, 120, 121]. For example, both Owen et al. [67] and Mallo and Dellal [55] showed increases in training intensity, measured via heart rate, to be associated with increased injury. Gabbett and Ullah [34] also found that when >9 m of sprinting (>7 m/s) per session was performed in elite rugby league players, this resulted in a 2.7-fold greater relative risk of sports performance non-contact, soft-tissue injury when compared with <9 m. In contrast to distances at sprinting velocity, it was found that sessions that had greater distances covered for very-low-intensity (0–1 m/s) and low-intensity running velocities (1–3 m/s) were associated with a reduced risk of time-loss non-contact injury. Low training intensity, such as that reported by Buist et al. [35] (i.e. “All were advised to run at a comfortable pace at which they could converse without losing breath”), may also account for increases in external training load of 23.7 %/week not being associated with increased injury versus 10 %/week increases. These lower intensities reported by Buist et al. and Gabbett et al. may have provided a recovery stimulus [8, 122] or allowed adaptation to occur without excessively fatiguing athletes so as to increase injury risk [116, 120].

#### Other Measures of Training Load

In addition to acute training load, other variables such as chronic load (previous 3- or 4-week total average load) [57, 85], monotony (total week load/SD of daily load) [74], strain (monotony × total week load) [74] and the acute:chronic workload ratio [57] may be more robust predictors of injury as they objectively account for accumulation of, and variability in, training load over time. As with acute load, both U-shaped and inverted-U-shaped relationships were present, along with positive and negative linear relationships for cumulative load. For example, Hulin et al. showed a linear protective effect for high chronic external load (previous 4-week average) [57], whereas Orchard et al. [58] showed higher 17-day external bowling loads (>100 overs) to increase injury risk 1.8-fold. Cross et al. [72] have also noted a U-shaped relationship with 4-week cumulative internal load, with an apparent increase in risk associated with higher internal loads (>8651 AU). In contrast, Colby et al. [85] found an inverted-U external load–injury relationship using 3-weekly total running distance; between 73 and 87 km was associated with 5.5-fold greater intrinsic (non-contact) injury risk in elite AF players when compared with low (<73 km) and high (>87 km) distances. The difference in patterns highlighted may be injury type-specific, as highlighted by Orchard et al. [59] in their review of the effects of cumulated load in 235 elite cricket fast bowlers over the longest period of study in the current literature (15 years). Previous 3-month load was found to be protective for tendon injury but injurious with respect to bone-stress injury. Increased previous season load was also associated with increased joint injuries but provided a protective effect for muscle injuries. Only one previous study found associations between illness and monotony and strain levels [74]. ‘Spikes’ in training monotony (>2.0) and strain levels were associated with rates of 77 and 89 %, respectively, in relation to illness [74]; however, no other studies reported any associations between injury/illness and monotony and strain levels [73, 106]. The results of our review have highlighted conflicting evidence for the use of monotony and strain. The weight of evidence favouring other metrics, such as change in acute training load [57, 72, 84, 87], and chronic training load [44, 45, 59] indicate that the role of monotony and strain in monitoring and injury prevention is not currently supported by the literature. A potential improvement on using acute and chronic load in isolation to predict injury is the acute:chronic workload ratio measure as it takes into account both acute and cumulative workload by expressing acute load relative to the cumulative load to which athletes are accustomed [57, 107]. The only study to use the acute:chronic workload ratio in this current review found that an acute:chronic ratio of 2.0, when compared with 0.5–0.99 for internal and external training load, was associated with 3.3- to 4.5-fold increased risk of non-contact injury in elite cricket fast bowlers [57].

### Fatigue Markers and Injury

Only nine studies investigated fatigue–injury relationships, seven of which used perceptual wellness scales [37, 39, 80, 92–95]. Three studies used the Hassles and Uplifts Scale (HUS) [123] and showed greater daily hassles to be associated with increased injury in soccer players [37, 92, 93]. Findings from Kinchington et al. [94] support the notion that increased perceptual fatigue is related to increased injury as ‘poor’ scores on the Lower-Limb Comfort Index (LLCI) [124] (i.e. an increase in perceptual fatigue) were related to increased lower-limb injury (*r* = 0.88; *p* < 0.001) in elite contact-sport athletes. Laux et al. [95] further support the positive perceptual fatigue–injury relationship in their findings, which reported that increased fatigue and disturbed breaks, as well as decreased sleep-quality ratings, were related to increased injury. In contrast, Killen et al. [80] found increased perceptual fatigue (measured via worse ratings of perceptual sleep, food, energy, mood, and stress) was associated with decreased training injury during an elite rugby league preseason (*r* = 0.71; *p* = 0.08). Similarly, King et al. [39] showed increased perceptual fatigue (measured via various REST-Q factors) was associated with decreased sports performance training injuries and time-loss match injuries. These unexpected findings may be due to the fact that when players perceive themselves to be less fatigued they may train/play at higher intensities, increasing injury likelihood [80]. Of the seven studies mentioned above, six used perceptual wellness scales that take approximately 1–4 min to complete. Shorter wellness scales, such as the 1–10 ratings used by Killen et al. [80], that have <1 min completion time may be easier to implement [125]; therefore, there is great practical significance in their association with injury. However, the differences in the levels of evidence for validation between psychometric tools and ‘bespoke’ wellness questionnaires are important factors when considering their use in an applied setting. The benefits of using REST-Q compared with shorter wellness scale questionnaires reflect the fact that REST-Q has undergone extensive tests of rigor, whereas the latter are not as well-validated [39, 139]. Examination of subjective fatigue markers also indicates that current self-report measures fare better than their commonly used objective counterparts [139]. In particular, subjective well-being typically worsened with an acute increase in training load and chronic training load, whereas subjective well-being demonstrated improvement when acute training load decreased [139]. Sleep is a vital part of the body’s recovery process [126, 127], therefore it was surprising that only four studies investigated its relationship with injury [39, 80, 95, 96]. Three studies assessed sleep–injury relationships via sleep-quality ratings [39, 80, 95], with only Dennis et al. [96] investigating objective measures of sleep quality and quantity in relation to injury. No significant differences in sleep duration and efficiency were reported between the week of injury and 2 weeks prior to injury.

### Individual Characteristics and Injury

An important finding from this review is that the individual characteristics of the athlete [36, 128] will significantly impact the internal load and stress placed on the body and thus the athlete’s susceptibility to injury. For example, an athlete’s aerobic fitness level will impact the internal workload they place on themselves. A recent study in AF players reported that for every 1 s slower on the 2-km time-trial performance, there was an increase in sRPE of 0.2. Therefore, the better the time trial performance of the individual, the easier the sessions of the same distances were rated [129]. Furthermore, older athletes or athletes with a previous injury are at a significantly greater risk of injury than other members of their population [27, 40, 84, 112, 130]. A potential reason for this finding is that it is likely older players have experienced a greater number of injuries across the course of their careers than the less experienced younger players [131]. Other individual characteristics, such as body composition, have a significant impact on injury. Zwerver et al. [128] reported a higher risk of RRI among persons with a body mass index (BMI) above 25 kg/m^2^, which is in agreement with Buist et al., who reported higher BMI scores in injured runners versus non-injured runners (BMI 27.6 vs. 24.8 kg/m^2^; *p* = 0.03) [35]. In addition, decreases in aerobic power and muscular power, as well as increases in skinfold thickness towards the end of the playing season, have been reported alongside increased match injury rates in recreational rugby league players [36]. Collectively, these findings suggest that the load/fatigue–injury associations described in this review are significantly influenced by the individual characteristics of the athlete, such as strength, fitness, body composition, playing level, age and injury history, as they determine the amount of internal workload and stress placed on the body and therefore the subsequent reduction or increase in the risk of injury [27, 35, 36, 40, 84, 112, 128–130].

### Training Load and Illness

Monitoring of training load and illness accounted for 17 of 68 studies in this review, with the majority measuring salivary immunoglobulin (Ig) A (s-IgA) and/or cortisol (*n* *=* 13) as a marker of immune function. The following section discusses the relationship between monitoring training load and key phases identified with increasing the susceptibility of the athlete to illness.

#### Intensification of Training Load and Illness

Internal training load, measured via sRPE, explained between 77 and 89 % of illness prevalence over a period of 6 months to 3 years in a mixed-ability group of swimmers [74]. Piggott et al. [68] identified that if weekly internal training load was increased by more than 10 %, this explained 40 % of illness and injury in the subsequent 7 days. This could be associated with elevated psychological stressors from increased internal training load and factors that were significantly associated with signs and symptoms of injury and illness [50]. Cunniffe et al. [54] reported that periods of increased training intensity and reduced game activity just prior to competition resulted in peaks in upper respiratory tract infection (URTI) in elite rugby union players. Despite the consensus that intensified periods in load or reduced game/training activity increased URTI, there was a contradiction in the literature as to whether increases in external load and markers of immune function were associated with the risk of illness [69, 97–99]. For example, Fricker et al. [69] found no significant differences in mean weekly and monthly running distances in elite male distance runners who self-reported illness versus those who did not. With the exception of Fricker et al. [69] and Veugelers et al. [70], the majority of studies that have found no association between load and illness have used mixed ability or disability populations, only measured external load, and used self-reported illness. Therefore, as the individual responses to load will vary dramatically with athlete training level, this may impact on illness rates. Furthermore, having athletes self-report illnesses rather than being diagnosed by a team doctor, and self-reporting load rather than having it measured objectively in terms of external load, may have a significant impact on the results (depending on the individual’s perception of what illness is and the potential for over- or underreporting of the amount of training exposure).

### Fatigue and Illness

Mackinnon and Hooper [49] found the incidence of URTI to be lower in athletes who reported increased perceptual fatigue via the 1–7 wellness rating scales (sleep quality, stress and feelings of fatigue). This study concurs with Killen et al. [80] who found lower injury rates in rugby league athletes who reported increased perceptual fatigue via perceptual wellness scales of 1–10. This is further supported by Veugelers et al. [70] who found that increased perceptual fatigue in their elite AF high internal training load group caused a protective effect against non-contact injury and illness when compared with the low internal training load group. This reduced injury/illness could be possibly due to greater fatigue resulting in a reduction in intensity, as a result of the physiological and psychological stress placed on the body. Another possibility is that the high internal training load group adapted to the load and were therefore able to tolerate higher internal load at a reduced risk of injury/illness. Finally, the lower training load associated with illness may be due to the fact that athletes could have had their training load modified as a result of being ill earlier in the week.

#### Markers of Immune Function and Illness

A primary finding was the association between s-IgA reduction and increased salivary cortisol due to periods of greater training intensities or reduced game/training activity (preseason, deload weeks), resulting in significant increases in URTI [54, 81, 83, 87, 90, 98, 101]. For example, rugby players who sustained a URTI, when compared with players who did not, had a reduction in s-IgA by 15 % [54]. However, there was contradiction within the literature on whether reduction in s-IgA was linked with URTI as Ferrari et al. [73] found no significant association between training load phase, s-IgA and sustained URTI in sub-elite male road cyclists. This is in agreement with Leicht et al. [99] who found secretion rate had no significant relationship with s-IgA responses and subsequent occurrence of upper respiratory symptoms in elite wheelchair rugby athletes.

### The Latent Period of Illness

A key phase identified with illness was the latency period, which can be defined as the time interval between a stimulus and a reaction [13]. At the onset of a stimulus, such as a spike in training load, reductions can occur in s-IgA or the elevation of salivary cortisol levels for an extended period of 7–21 days. Failure of these markers to return to baseline values during this time period was associated with a 50 % increased risk of URTI [68, 90, 100], which would explain why the majority of illnesses reported in this review occurred during or after week 4 of intensified training [81, 83, 90, 100, 101]. Athletes who do not recover from the initial spike in training load experience an extended period of suppression of immune function, placing the athlete at a significant risk of illness. This finding has implications for practitioners; first, to avoid unplanned spikes in training load and, second, to adjust training loads when an athlete is immunosuppressed to allow the markers of immune system to return to baseline values.

### Limitations

Of the 68 studies included in this review, 39 were only highlighted from the search criteria, with an additional 29 included from searching references of the identified studies, which could have led to the risk of studies not being included. Furthermore, during the manuscript review process, a number of key studies and reviews were published that would have satisfied the inclusion criteria and provided the most up-to-date research [13, 131, 144, 146, 148]. For example, several papers readdressed terminology issues in relation to use of the training stress balance measure, and defined it as the acute:chronic workload ratio [108, 143, 145, 146, 148].

### Directions for Future Research

#### Definition of Load, Fatigue, Injury and Soreness

Even with the relatively small amount of research undertaken regarding longitudinal monitoring, the research detailed in this review clearly highlights that relationships exist between longitudinally monitored training load and fatigue variables and injury or illness. Further research is now required to establish a common language for load, fatigue, injury and illness, as well as exploring these relationships within more specialised populations, and with a wider range of load, fatigue, injury and illness measures.

#### Training Load and Fatigue Interactions

A clear gap identified in the literature from the current review is the lack of assessment of load–fatigue interactions in association with injury/illness, as the fatigue state of an individual will essentially define the load they can tolerate before injury/illness risk increases [121, 132]. For example, a case study on a female masters track and field athlete found that, despite no increase in load, signs of overreaching increased significantly due to external psychological stress [133]. Therefore, further study is needed combining both load and fatigue in analyses, as per the study by Main et al. [50].

#### Monitoring of Neuromuscular Function

The lack of monitoring of NMF variables in respect to injury and illness (*n* *=* 0 of the 68 studies included within this review) was highly surprising given the strong theoretical rationale for its association with injury risk [120, 134] and its common use in the recovery and acute monitoring literature [7, 135, 136] in light of its strong association with performance variables such as speed [137]. Future research should investigate the relationship between NMF variables and training load, and their consequent associations between injury and illness; however, there is currently no high-level evidence to support its use in monitoring as mechanism-based reasoning represents the lowest form of evidence [151].

#### Perceptual Wellness

Of the 13 studies that used perceptual wellness measures, 11 adopted inventories that take approximately 1–4 min to complete. In a busy elite athlete environment or, conversely, a recreational/sub-elite environment where resources are stretched, inventories of such length may be impractical to implement [125]. Shorter wellness scales, such as the 1–10 rating scales used by Killen et al. [80] and the 1–7 rating scales used by MacKinnon and Hooper [49], that have <1 min completion time may be easier to implement [125]. Consequently, more investigation is needed using shortened perceptual wellness scales as there is great practical significance in their association with injury; however, these should undergo suitable tests of their validation in order to ensure they are able to detect the intended domains and constructs in a rigorous manner.

#### Latent Period of Injury

This review highlighted a lack of studies reporting the latency period of injury. Future studies evaluating the relationship between training load and time frame of injury response will provide information that allows practitioners to adjust training loads during the injury time frame as an injury prevention measure [149].

#### Injury and Illness

Monitoring of load–fatigue and injury and illness accounted for only 6 of the 68 studies in this review. Although studies investigating both injury and illness accounted for <10 % of the research in this review, the majority (five of six) were above the average quality score (13 vs. 11) (Table 3). Future research should therefore look to measure both injury and illness, not only because of the higher quality scores accorded to studies that did so in this review but also because of the different relationships they will highlight between load and fatigue markers and subsequent injury risk and performance outcomes [29, 110, 138].

#### Monitoring of Female Athletes

This review has highlighted a lack of studies with female athletes (13 of 68 studies). Further research is essential to understand how the hormonal fluctuations during various stages of the menstrual cycle may influence tolerance to training load, and the subsequent effects on markers of immune function and fatigue markers. This will provide valuable information on load and fatigue and inform the periodization of female athletes to help reduce the risk of injury/illness during periods of greater susceptibility.

#### Monitoring of Adolescent Athletes

Although this review has highlighted a lack of studies in adolescents, a recent review found that the relationship between workload, physical performance, injury and illness in adolescent male football players was non-linear and that the individual response to a given workload is highly variable [150]. Further investigation into the effects of maturation and training loads, and their relationship between performance, injury and illness, would be invaluable for practitioners working with pediatric athletes.

#### Session Rate of Perceived Exertion

The widespread use of sRPE as a measure of internal training load is most likely due to its relative ease of implementation compared with other internal load measures, such as heart rate or external load measures from GPS systems [17]. Indeed, a recent review has highlighted that current self-report measures fare better than their commonly used objective counterparts [139]. To advance the use of self-reported measures, splitting sRPE into internal respiratory and muscular load is also warranted to observe how such discrepancies affect injury/illness [140, 141]. The injury/illness mechanisms will differ between these two systems and such differential measurement of internal load will allow more specified information for prevention and recovery [140].

#### Severity of Injury

Only a small number of studies in this review investigated the severity of injury [38, 41, 45–47, 67, 77] and illness [75, 82], with only two studies quantifying the contact elements of training/competition [64, 65]. Given contact injuries are often more severe than non-contact injuries [41], and the amount of time lost from training/competition is one of the major negative impacts of injury/illness, more studies are needed detailing load/fatigue–injury/illness severity and the contact aspects of training/competition. Information on injury/illness severity relationships will allow coaches and support staff to make even more informed decisions about the risk of going beyond thresholds of load/fatigue, such that they may accept an increased risk of sports performance or low-severity injuries, but not accept increases in the risk of more severe injuries.

## Conclusions

This paper provides a comprehensive review of the literature that has reported the monitoring of longitudinal training load and fatigue and its relationship with injury and illness. The current findings highlight disparity in the terms used to define training load, fatigue, injury and illness, as well as a lack of investigation of fatigue and training load interactions. Key stages of training and competition where the athlete is at an increased risk of injury/illness risk were identified. These included periods of training load intensification, accumulation of training load and acute change in load. Modifying training load during these periods may help reduce the potential for injury and illness. Measures such as acute *change* in training load, cumulative training load, monotony, strain and acute:chronic workload ratio may better predict injury/illness than simply the use of acute training load. Acute change in training load showed a clear positive relationship with injury, with other load/fatigue measures producing mixed associations, particularly acute and cumulative training load. The measure most clearly associated with illness was s-IgA, while relationships for acute training load, monotony, strain and perceptual wellness were mixed. The prescription of training load intensity and individual characteristics (e.g. fitness, body composition, playing level, injury history and age) may account for the mixed findings reported as they impact the internal training load placed on the athlete’s body and, therefore, susceptibility to injury/illness.
